# Revealing the Potential of Lipid and β-Glucans Coproduction in *Basidiomycetes* Yeast

**DOI:** 10.3390/microorganisms8071034

**Published:** 2020-07-13

**Authors:** Dana Byrtusová, Volha Shapaval, Jiří Holub, Samuel Šimanský, Marek Rapta, Martin Szotkowski, Achim Kohler, Ivana Márová

**Affiliations:** 1 Faculty of Chemistry, Brno University of Technology, Purkyňova 464/118, 612 00 Brno, Czech Republic; jirka.hollub@seznam.cz (J.H.); samuell.sim@hotmail.com (S.Š.); xcrapta@fch.vut.cz (M.R.); xcszotkowski@fch.vut.cz (M.S.); marova@fch.vut.cz (I.M.); 2 Faculty of Science and Technology, Norwegian University of Life Sciences, Postbox 5003, 1432 Ås, Norway; volha.shapaval@nmbu.no (V.S.); achim.kohler@nmbu.no (A.K.)

**Keywords:** red yeast, β-glucans, lipids, high-throughput screening, carbon:nitrogen ratio, osmotic stress

## Abstract

Beta (β)–glucans are polysaccharides composed of D-glucose monomers. Nowadays, β-glucans are gaining attention due to their attractive immunomodulatory biological activities, which can be utilized in pharmaceutical or food supplementation industries. Some carotenogenic *Basidiomycetes* yeasts, previously explored for lipid and carotenoid coproduction, could potentially coproduce a significant amount of β–glucans. In the present study, we screened eleven *Basidiomycetes* for the coproduction of lipids and β–glucans. We examined the effect of four different C/N ratios and eight different osmolarity conditions on the coproduction of lipids and β–glucans. A high-throughput screening approach employing microcultivation in microtiter plates, Fourier Transform Infrared (FTIR) spectroscopy and reference analysis was utilized in the study. Yeast strains *C. infirmominiatum* CCY 17-18-4 and *R. kratochvilovae* CCY 20-2-26 were identified as the best coproducers of lipids and β-glucans. In addition, *C. infirmominiatum* CCY 17-18-4, *R. kratochvilovae* CCY 20-2-26 and *P. rhodozyma* CCY 77-1-1 were identified as the best alternative producers of β-glucans. Increased C/N ratio led to increased biomass, lipid and β-glucans production for several yeast strains. Increased osmolarity had a negative effect on biomass and lipid production while the β-glucan production was positively affected.

## 1. Introduction

Recently, coproduction strategies, when two or more valuable products are simultaneously produced in a single fermentation process, have been proposed as a way to reach sustainable production of microbial biomass for a wide range of applications [1]. When developing a coproduction process, it is important to identify suitable microbial cell factories able to perform concomitant production of several metabolites and optimize cultivation conditions for the most optimal and balanced biosynthesis of all the desired products.

Carotenogenic *Basidiomycetes* could be one of the promising microorganisms used for developing coproduction processes. Carotenogenic *Basidiomycetes* can be enriched in lipids (up to 70% *w*/*w*) [2], carotenoids [3], ergosterol [4] and glucans [5,6]. Currently, there is one main coproduction strategy for *Basidiomycetes* presented in the literature, and it is related to the coproduction of lipids and carotenoids [3,7]. This strategy has several limitations related to the separation of lipophilic components in the case of their separate use [8]. The strategy of coproduction of lipids and β–glucans was not previously revealed, while it has a significant advantage over the lipid−carotenoid coproduction. Since β–glucans are located within the cell wall, which is the leftover material after lipid extraction, they, therefore, could be easily separated.

According to the report by Business Communications Company (BCC) Research, the global β-glucan market is expected to reach 576.28 million USD by 2025, growing at a compound annual growth rate (CAGR) of 7.3% from 2017 to 2025 [9]. The increasing demand for β-glucans requires the search for alternative sources. While mushrooms and plants were extensively explored, yeast sources of β-glucans were limited to the baker’s yeast *Saccharomyces cerevisiae.* The cell wall of *Saccharomyces cerevisiae* contains two types of β-glucans: the major component (50–55% of the cell wall) is represented by linear *β*-1,3-D-glucan and the other type is branching *β*-1,6-D-glucan (10–15%) [10]. Production of yeast β-glucans shows a higher yield and the extraction process is more economically efficient than in the case of plant β-glucans [11,12]. Therefore, there is a strong need to identify other alternative yeast sources suitable for the production of β-glucans.

Carotenogenic *Basidiomycetes* yeast represents a biotechnologically unexplored potential source of β-glucans. Carotenogenic *Basidiomycetes* yeasts of the genera *Rhodotorula, Cystophilobasidium, Sporobolomyces*, and *Phaffia* have been extensively studied as an alternative source of lipids and carotenoids [13], while the research performed on their potential as a source of β-glucans is very limited [5]. The ability of these yeasts to simultaneously accumulate lipids and potentially β-glucans calls for the development of a unique coproduction concept when both lipids and β-glucans are produced in a single fermentation process. Further, it is important to note that *Basidiomycetes* lipids are rich in saturated and monounsaturated fatty acids and, therefore, considered as low-value lipids with the application mainly for biodiesel production and/or as animal feed ingredients. Thus, the coproduction of β–glucans and lipids could significantly increase the value of *Basidiomycetes* lipid production, and the biomass itself.

The aim of this study was to perform, for the first time, a high-throughput screening of eleven *Basidiomycetes* strains, for identifying the best coproducers of lipids and β-glucans. In the study we used two control strains, *Saccharomyces cerevisiae*, identified as the best producer of β-glucans [10] and *Rhodotorula toruloides*, identified as the best lipid producing yeast [2]. In addition, three *Metchnikowia* strains were included in the study as potential lipid accumulating *Ascomycetes* yeasts [14]. As optimization parameters, four carbon:nitrogen (C/N) ratios and eight different extracellular osmolarity conditions were selected in total. The screening cultivation was performed in the high-throughput Duetz Microtiter Plate System (Duetz-MTPS) allowing reproducible cultivations which have been shown to be scalable to shake flasks and fermenter-type cultivations [15]. In addition, high-throughput Fourier transform infrared (FTIR) spectroscopy was utilized for total cellular biochemical profiling of differently cultivated *Basidiomycetes* and *Ascomycetes* yeasts [16,17,18,19,20,21].

## 2. Materials and Methods

### 2.1. Yeast Strains

Eleven carotenogenic *Basidiomycetes* yeast strains belonging to the genera *Cystofilobasidium*, *Rhodotorula*, *Sporidiobolus*, and *Phaffia*, and four *Ascomycetes* noncarotenogenic yeast strains belonging to genera *Metchnikowia* and *Saccharomyces* obtained from the Culture Collection of Yeasts (Institute of Chemistry, Slovak Academy of Science, Bratislava, Slovakia) were used in the study. *Saccharomyces cerevisiae* was used as a control strain for β-glucan production, while *Rhodotorula toruloides* was used as a control strain for lipid production. The detailed list of yeast strains is presented in Table 1.

### 2.2. Media and Growth Conditions

The cultivation of yeast was performed first on YPD agar medium (Merck, Darmstadt, Germany) to recover frozen cultures and then in YPD broth medium (Merck, Darmstadt, Germany) without nutrient limitation to prepare inoculum. For the screening study, the production broth media (Merck, Darmstadt, Germany) with different C/N ratios (10:1, 40:1, 70:1 and 100:1) were inoculated.

For the YPD agar cultivation, yeast cells from the frozen cryopreserved stock cultures were transferred onto Petri dishes with YPD agar (yeast extract, 10.0 g/L; peptone, 20.0 g/L; glucose 20.0 g/L; agar, 20.0 g/L) (Merck, Darmstadt, Germany) and cultivated for 72 h at 25 °C. Inoculum was prepared by transferring 1µL of yeasts cells from YPD agar into 50 mL of sterile YPD broth medium (yeast extract, 10.0 g/L; peptone, 20.0 g/L; glucose 20.0 g/L) (Merck, Darmstadt, Germany) in Erlenmeyer flask (250 mL) and cultivated for 24 h at 25 °C under shaking regime (100 rpm, 50 mm). To remove the residual medium after the cultivation, the inoculum cells were washed with the sterile 0.1% (*w*/*v*) NaCl and resuspended to the original volume. The inoculum was added in the volume ratio of 1:5 to the YPD production broth media with different C/N ratios, which was composed of (g/L): yeast extract, 2; KH_2_PO_4_, 4; MgSO_4_ · 7H_2_O, 0.7 and glucose monohydrate. The following C/N ratios were examined, 10:1, 40:1, 70:1 and 100:1. The carbon content of 40% in glucose and nitrogen content of 10.5% in yeast extract was used for the calculation of the C/N ratios. The cultivation in production media was performed in a Duetz Microtiter Plate System [15,22,23] (Enzyscreen, Heemstede, Netherlands) which consists of 24-well extra deep microtiter plates (MTPs) with low-evaporation sandwich covers and clamp system for mounting MTPs onto the incubator shaking platform. Cultivation in the control YPD broth production media was done for 96 h at 25 °C according to Szotkowski et al. (2019) under the shaking regime (100 rpm, 50 mm) [6].

For the investigation of the influence of increasing osmolarity on the simultaneous production of glucans and lipids, four yeast strains with high β-glucan production, namely *Saccharomyces cerevisiae* (CCY 21-4-102), *Cystofilobasidium infirmominiatum* (CCY 17-18-4), *Phaffia rhodozyma* (CCY 77-1-1) and *Rhodotorula kratochvilovae* (CCY 20-2-26) were selected. The media with C/N ratios 40:1, 70:1 and 100:1 were supplemented with NaCl to final concentrations of 0.2, 0.5, 2, 5, 8 and 11% (*w*/*v*). All cultivation media were sterilized at 121 °C for 15 min and the biomass was harvested after 96 h of cultivation.

### 2.3. Experimental Design

Three biological replicates were prepared for each strain and cultivation medium by inoculating strains from frozen cryopreserved stocks onto agar plates. Each biological replicate was prepared in a separate microtiter plate. All microtiter plates (MTPs) were prepared by inoculating three wells for each strain and cultivation medium. At the end of cultivation, cell suspensions of the three wells were merged and the final biomass was used for total biochemical profiling by FTIR spectroscopy, lipid analysis by gas chromatography and glucan analysis by Yeast and Mushroom β-glucan Assay Procedure (486 samples in total). Morphology of some yeast strains was additionally examined by optical microscope Leica DM6 B (Leica Microsystems, Wetzlar, Germany).

### 2.4. Preparation of Yeast Biomass for the Glucan and Lipid Analysis

After cultivation, the yeast biomass was centrifuged at 4500 rpm for 5 min at 4 °C and the biomass pellet was then washed three times using 0.1% NaCl solution. Then the yeast biomass was freeze-dried for 48 h and subsequently stored at −20 °C until use.

### 2.5. Analysis of Glucans by Yeast and Mushroom β-Glucan Assay

The total glucan content, and the content of β- and α-glucans were determined according to the Yeast and Mushroom β-glucan Assay Procedure K-YBGL (Megazyme Int., Warszawa, Poland) [24,25,26]. To estimate the total glucan content, freeze-dried yeast biomass was hydrolysed with ice-cold 12 M sulphuric acid for 2 h and then incubated for 2 h at 100 °C. Further, acidic hydrolysate was neutralized with 200 mM sodium acetate buffer (pH 5) and 10 M KOH, followed by the effect of enzymes exo-β-(1→3)-D-glucanase and β-glucosidase in acetate buffer (pH 4.5) for 1 h at 40 °C. The α-glucan content was determined after enzymatic hydrolysis with amyloglucosidase and invertase. The β-glucan content was calculated from the assay kit procedure as the difference between total glucan and α-glucan content. The absorbance values indicating the total glucans and α-glucan content were obtained spectrophotometrically at 510 nm after adding glucose oxidase/peroxidase reagent.

### 2.6. Total Lipid Content and Analysis of Fatty Acid Profile

Lipid extraction was performed by a modified Folch method [22,23]. A sample of 15 ± 3 mg of freeze-dried yeast biomass was added into 2 mL polypropylene (PP) tubes together with 250 ± 20 mg acid-washed glass beads (710–1180 μm diameter, Millipore Sigma, St. Louis, Missouri, USA) and 600 μL methanol. For the disruption of yeast cells, the Precellys evolution homogenizer (Bertin Instruments, Germany) was used three times with shaking cycles of 5500 rpm (2 × 20 s). The content of the PP tube was transferred into a glass reaction tube by washing it with a 2.4 mL solvent mixture of methanol:chloroform:hydrochloric acid (7.6:1:1 *v*/*v*). A 1 mg dose of tridecanoid acid (C13:0) in the form of triacylglycerol (TAG) was used as an internal standard and added to the reaction mixture. The glass tube was vortexed for 10 s and incubated for 1 h at 90 °C. After cooling to room temperature, 1 mL of distilled water and 2 mL hexane:chloroform (4:1 *v*/*v*) mixture were added. The separated upper hexane phase with extracted lipids was evaporated under nitrogen at 30 °C followed by the addition of sodium sulfate and dissolving the fatty acid methyl esters (FAMEs) in 1.5 mL hexane containing 0.01% butylated hydroxytoluene (BHT, Millipore Sigma, St. Louis, Missouri, USA). The hexane containing the extracted lipids was transferred into glass vials for GC analysis. Total lipid content (wt.% of total FAMEs of the dry weight) and the fatty acid profile were estimated by 7820A gas chromatograph, Agilent Technologies equipped with an Agilent J&W GC column (20.0 m × 180 μm × 0.20 μm) and flame ionization detector (FID). The total time of the analysis was 36 min with an initial temperature of 70 °C, which was kept for 2 min, and then increased by 10 °C/min to 150 °C, and finally by 6 °C/min to 230 °C. A dose of 1 μL of the sample was injected in split mode (30:1 split ratio) to an inlet tempered to 280 °C. FAME standard mixture (C4–C24; Millipore Sigma, St. Louis, MO, USA) dissolved in hexane was used for the identification of the FAMEs. Quantification was based on the C13:0 internal standard.

### 2.7. Total Lipid Content and Analysis of Fatty Acid Profile

The biomass of the yeasts grown on different C/N ratios was subjected to the biochemical profiling by FTIR spectroscopy. Washed yeast suspension (4 μL) was transferred on 384-well ZnSi microplate (Bruker Optik, Ettlingen, Germany) in triplicate. Samples were dried at room temperature for 30 min before analysis. FTIR spectra were recorded in transmission mode using the High Throughput screening eXTension unit (HTS-XT) coupled to the Vertex 70 FTIR spectrophotometer (Bruker Optik, Ettlingen, Germany). The spectra were collected in the spectral range from 4000 to 500 cm^−1^ (spectral resolution of 6 cm^−1^, and aperture 5.0 mm), with 64 scans as average for each sample.

### 2.8. Data Analysis

Prior to principle component analysis (PCA), the FTIR spectra were preprocessed. The preprocessing was performed by transforming spectra to the second-derivative using the Savitzky−Golay algorithm with a polynomial of degree 2 and a window size of 11. The second-derivative spectra were preprocessed by extended multiplicative signal correction (EMSC) [27,28,29]. Technical replicates (543 spectra in total) were averaged in order to remove technical variability of the measurements, resulting into 181 spectra. PCA was performed for three spectral regions, lipid (3050–2800 cm^−1^ combined with 1800–1700 cm^−1^), protein (1700–1500 cm^−1^) and polysaccharide (1200–700 cm^−1^).

The following software packages were used for the data analysis: Unscrambler X version 10.5.1 (CAMO Analytics, Norway), Orange data mining toolbox version 3.24 (University of Ljubljana, Slovenia) [30,31].

## 3. Results

### 3.1. Growth, Total Glucan and β-Glucan Content in Basidiomycetes Yeast

**Figure 1:**
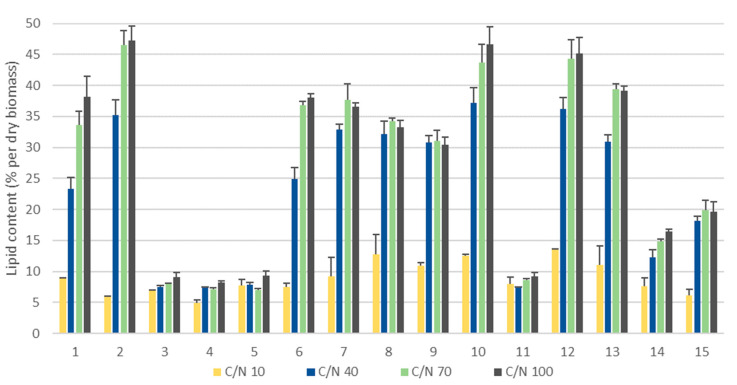
Lipid content (% *w*/*w*) in yeast grown in the media with low and high C/N ratios. Yeast strain numbers are described in Table 1.

**Figure 2:**
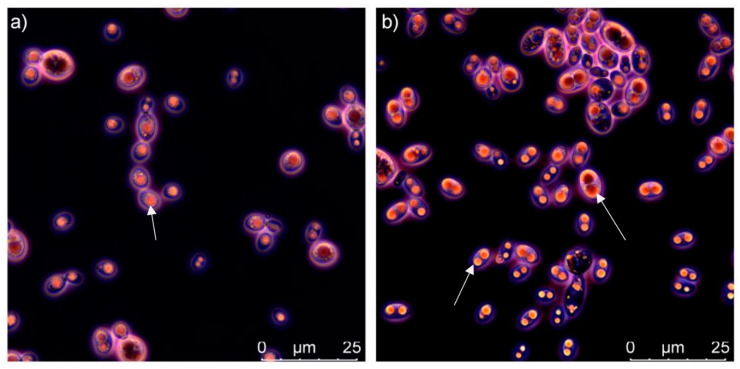
Microscopy images (false coloring) of C. infirmominiatum CCY 17-18-4 (**a**), and C. macerans CCY 10-1-2 (**b**) grown in medium with C/N ratio 100:1 (white arrows indicate lipid droplets).

**Figure 3:**
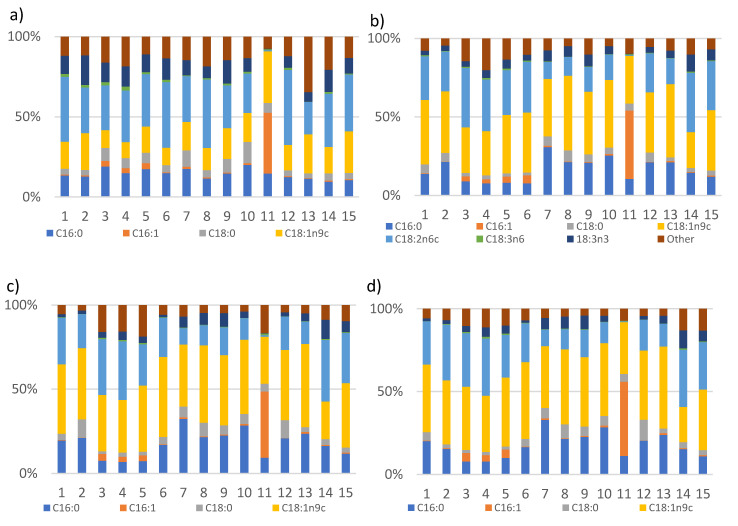
Fatty acid profile of TAGs accumulated in yeast grown in the media with (**a**) C/N 10:1, (**b**) C/N 40:1, (**c**) 70:1, (**d**) C/N 100:1 ratio.

**Figure 4:**
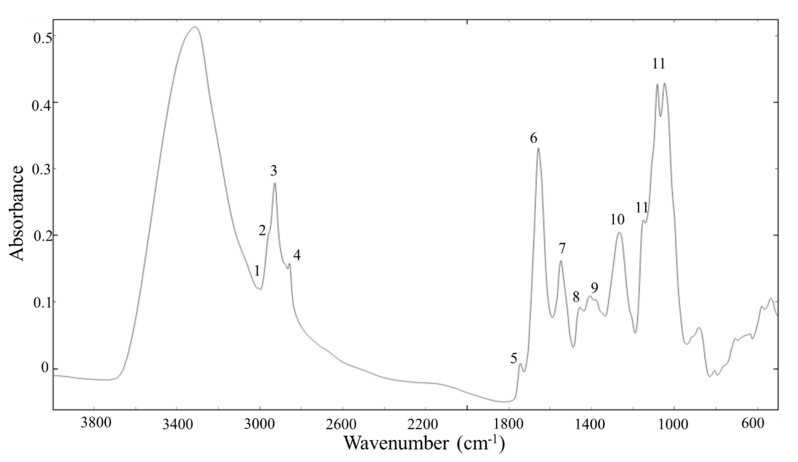
Preprocessed FTIR spectra *S. cerevisiae* CCY 21-4-102 biomass grown in the C/N ratio 70:1. Peak numbers correspond to the numbers given in Table 4.

The revealing of potential coproducers of lipids and β-glucans and identifying of new promising yeast sources of β-glucans were based on estimating the following parameters: biomass yield, total glucans, β- and α-glucans, lipid yield and lipid profile (Table 2, Table 3, Table 4, Table 5, Table 6 and Table 7, Figure 1, Figure 2, Figure 3 and Figure 4). Cultivation of yeasts in the media with low (10:1), moderate (40:1) and high (70:1 and 100:1) C/N ratios showed a continuous increase in a biomass yield with the increase of C/N ratio (Table 2). Thus, for most of the studied yeast strains the highest biomass yield was in a range from 2.67 ± 0.26 to 15.33 ± 1.16 g/L and observed in a medium with a C/N ratio of 100. Yeast strains *R. kratochvilovae* CCY 20-2-26, *R. mucilaginosa* CCY 19-4-6 and CCY 20-9-7, *R. toruloides* CCY 62-2-4 and *S. pararoseus* CCY 19-9-6 showed the highest biomass production in a medium with a C/N ratio of 70:1 (Table 2). Generally, *Ascomycetes* yeasts showed lower biomass production in comparison to carotenogenic *Basidiomycetes* yeast (Table 2). The highest biomass yield 15.3 g/L was obtained for the strain *C. macerans* CCY 10-1-2 at a C/N ratio of 100. The biomass yield was nearly three times higher than for the reference β-glucan producing strain *Saccharomyces cerevisiae* CCY 21-4-102. For all *Rhodotorula* strains and *S. salmonicolor* CCY 19-4-25, a significant increase in biomass production occurred in the C/N ratio range from 10:1 to 40:1, and from 70:1 to 100:1, while little change was observed in the C/N ratio range from 40:1 to 70:1.

We observed an increase in the glucan and lipid content at high C/N ratio for the yeast strains showing an increase in biomass yield (Table 3, Figure 1), with the exception of *C. macerans* CCY 10-1-2, *C. infirmominiatum* CCY 17-18-4, *S. metaroseus* CCY 19-6-20 in the case of glucans and *S. salmonicolor* CCY 19-6-4 in the case of lipids. For the strains *C. macerans* CCY 10-1-2, *P. rhodozyma* CCY 77-1-1 and *R. toruloides* CCY 62-2-4, the highest total glucan content was detected at low C/N ratios (Table 3). While the highest β-glucan content 26.96 ± 2.90% (*w*/*w*) was achieved for the control strain *S. cerevisiae* under a C/N ratio of 100, the comparable total glucan content of 30.15 ± 3.21 (*w*/*w*) with the high content of β-glucans 25.34 ± 3.79 (*w*/*w*) was recorded for *C. infirmominiatum* CCY 17-18-4 (Table 3). Several other yeast strains showed similarly high β-glucan content at different C/N ratios: (i) at a C/N ratio of 10, strains *P. rhodozyma* CCY 77-1-1, *S. metaroseus* CCY 19-6-20, *R. kratochvilovae* CCY 20-2-26 and *C. macerans* CCY 10-1-2 had a β-glucan content between 21.68% and 24.52% (*w*/*w*); (ii) at 40:1 C/N ratio, strain *C. infirmominiatum* CCY 17-18-4 accumulated 25.34 ± 3.79% (*w*/*w*) of β-glucans; (iii) at a C/N ratio of 70:1, strain *M. pulcherrima* CCY 29-2-149 accumulated 20.45 ± 0.86% (*w*/*w*) of β-glucans and (iv) at a C/N ratio of 100, strains *M. pulcherrima* CCY 29-2-129 and *M. pulcherrima* CCY 29-2-147 showed β-glucan content of 22.35 ± 1.68% (*w*/*w*) and 21.09 ± 1.68% (*w*/*w*), respectively (Table 3). Taking into account the high biomass yield and β-glucan content for some of the yeast strains, as for example, *C. infirmominiatum* CCY 17-18-4 (3.15 g/L of β-glucans at C/N 100:1), *R. kratochvilovae* CCY 20-2-26 (2.58 g/L of β-glucans at C/N 70:1) and *P. rhodozyma* CCY 77-1-1 (2.43 g/L of β-glucans at C/N 100:1), they could be considered as promising new alternative yeast sources of the β-glucans. The yield of 1.60 g/L of β-glucans was present at *S. cerevisiae* CCY 21-4-102 at C/N 100:1.

Most of the studied yeast strains showed a negligible presence of α-glucans, while some of them had a relatively high content (Table 3). Interestingly the highest α-glucan content, that was over 5% per dry weight, was observed for the strain *C. macerans* CCY 10-1-2, which exhibited one of the lowest β-glucan content. Meaning that the ratio of α- and β-glucans in this strain was 1:2 at a C/N ratio of 100, while in all other yeast strains β-glucans were in a significantly higher proportion than α-glucans (Figure 1). The second highest content of α-glucans (4.81 ± 1.81% per dry weight) was observed for *C. infirmominiatum* CCY 17-18-4 which also had the highest content of β-glucans (25.34 ± 3.79% CDW) (Table 3) from all the studied carotenogenic yeasts. A relatively high content of α-glucans was measured for strains from genus *Sporidiobolus* and strain *P. rhodozyma* CCY 77-1-1 (Table 3) as well, where the total glucan content increased at higher C/N ratios. The lowest α-glucan content was detected for yeasts of *Metschnikowia* genera and *R. mucilaginosa* strains, where it did not exceed 1.5% of the total dry weight (Table 3).

### 3.2. Coproduction of Lipids and β-Glucans in Basidiomycetes Yeast

Basidiomycetes carotenogenic red yeasts showed oleaginous properties and were able to accumulate lipids from 30 to over 47% (*w*/*w*) while *Ascomycetes* noncarotenogenic yeast did not accumulate lipids more than 10% (*w*/*w*) (Figure 1). In the present study the noncarotenogenic *Metschnikowia* did not accumulate more than 10% (*w*/*w*). Generally, *Ascomycetes* yeasts were not affected by the variation in the amount of glucose in the media, and the biomass and lipid yield were unchanged.

High lipid yield in a range from 33 to 47% (*w*/*w*) was observed for the strains *C. infirmominiatum* CCY 17-18-4, *C. macerans* CCY 10-1-2, *R. toruloides* CCY 62-2-4, *S. metaroseus* CCY 19-6-20 and *S. pararoseus* CCY 19-9-6 when grown under high C/N ratios. The highest lipid content 47.27 ± 2.36% (*w*/*w*) was observed for the carotenogenic strain *C. macerans* CCY 10-1-2 (Figure 1). The confocal light microscopy of *C. macerans* CCY 10-1-2 cells showed clearly visible large round structures: lipid droplets (Figure 2). Yeast strains with the highest lipid accumulation (over 45% *w*/*w*), namely *C. macerans* CCY 10-1-2, *R. toruloides* CCY 62-2-4 and *S. metaroseus* CCY 19-6-20 showed low β-glucan content (10–14% *w*/*w*) and can be considered as mainly lipid producers. While some yeast strains as for example *C. infirmominiatum* CCY 17-18-4 at 100:1 C/N and *R. kratochvilovae* CCY 20-2-26 at 70:1 C/N were able to accumulate a relatively high content of both lipids (38.21 and 37.92% of *w*/*w*) and β-glucans (20.73 and 21.43% of *w*/*w*) accompanied by a high biomass yield (15.19 and 12.05 g/L) and therefore could be utilised for developing a strategy of lipid and β-glucan coproduction (in our experiment up to 3.15 g/L of β-glucans for *C. infirmominiatum* CCY 17-18-4).

### 3.3. Coproduction of Lipids and β-Glucans in Basidiomycetes Yeast

Lipids accumulated in yeasts are represented mainly by triacyl glycerides (TAGs). A detailed fatty acid profile was identified by gas chromatography, where fatty acids present in amounts higher that 2% were taken into consideration. In all the yeast strains the major saturated fatty acids were palmitic (C16:0) and stearic acid (C18:0); monosaturated, palmitoleic (C16:1) and oleic acid (C18:1n9c). Polyunsaturated fatty acids were represented by linoleic (C18:2n6c), γ-linolenic (C18:3n3) and α-linolenic (C18:3n3) fatty acids (Figure 3). The most abundant fatty acid present in all the yeast strains was oleic acid, with a production of over 40% in all strains except for *S. cerevisiae* CCY 21-4-102, where the major fatty acid was palmitoleic fatty acid. Depending on the C/N ratio and yeast strain, the content of monounsaturated (MUFA), polyunsaturated (PUFA) and saturated (SAT) fatty acids changed significantly (Figure 3). The content of MUFA, specifically oleic fatty acid, increased with the increase in C/N ratio in all yeast strains (Figure 3). The highest amount of MUFA (49% *w*/*w*) was detected for the strain *S. metaroseus* CCY 19-6-20, which showed also one of the highest total lipid and biomass yields (Table 2, Figure 1). The biggest difference in fatty acid profile can be observed for C/N ratios between 10:1 and 100:1, where the highest SAT content in all yeasts was detected at a C/N ratio of 10:1 (Figure 3) while higher C/N ratios favoured oleic acid biosynthesis and suppressed linoleic fatty acid production (Figure 3). Interestingly, the fatty acid profile of strains from genus *Metschnikowia,* belonging to the *Ascomycetes* phylum, was more similar to the fatty acid profile of carotenogenic *Basidiomycetes* yeast. It differed only by a slightly higher production of C16:1 and lower production of C16:0 (Figure 3). Among the studied *Basidiomycetes* yeasts, *Rhodotorula* yeasts showed a high production of palmitic fatty acid (Figure 3). The strain *S. cerevisiae* CCY 19-6-4 showed a very consistent fatty acid profile which was not affected by the different C/N ratios, which had the lowest production of PUFAs and the highest amount of palmitoleic fatty acid (C16:1) at all studied C/N ratios (Figure 3). The highest content of PUFA, with linolenic fatty acid dominating, was detected in the strain *S. salmonicolor* CCY 19-6-4 with a yield of up to 40% *w*/*w* at a C/N ratio of 40:1 (Figure 3).

### 3.4. Total Cellular Biochemical Profiling by FTIR Spectroscopy

The obtained FTIR spectra at different spectral regions provided information on all the main biochemical building blocks of the yeast cells. Lipids were described mainly by the two spectral regions 3010–2800 cm^−1^ and 1800–1700 cm^−1^ and some single peaks related to -CH_2_ and -CH_3_ scissoring in a region 1400–1300 cm^−1^ (Table 4, Figure 4). When analyzing cellular lipid profiles based on FTIR spectra, the most important lipid associated peaks usually taken into consideration are the following: (1) the peaks 2947 cm^−1^, 2925 cm^−1^, 2855 cm^−1^, 1465 cm^−1^ and 1377 cm^−1^ are related to -CH_3_ and -CH_2_ stretching and indicating mainly the chain length of the carbon skeleton in lipid molecules; (2) the peak 1745 cm^−1^ is related to the ester bond stretching and indicates the total lipid content in the cell; (3) the peak 1710 cm^−1^ is related to the carboxyl bond vibrations in free fatty acids, and (4) the peak 3010 cm^−1^ is related to = C-H stretching in lipids and indicates the unsaturation level of cellular lipids. Proteins were observed in the spectral region 1700–1500 cm^−1^ with the main peaks for amide I (1650 cm^−1^) and amide II (1540 cm^−1^) bonds and polysaccharides were observed in the region 1200–900 cm^−1^ which was mainly related to the sugar ring vibrations (Table 4, Figure 4).

**Figure 5:**
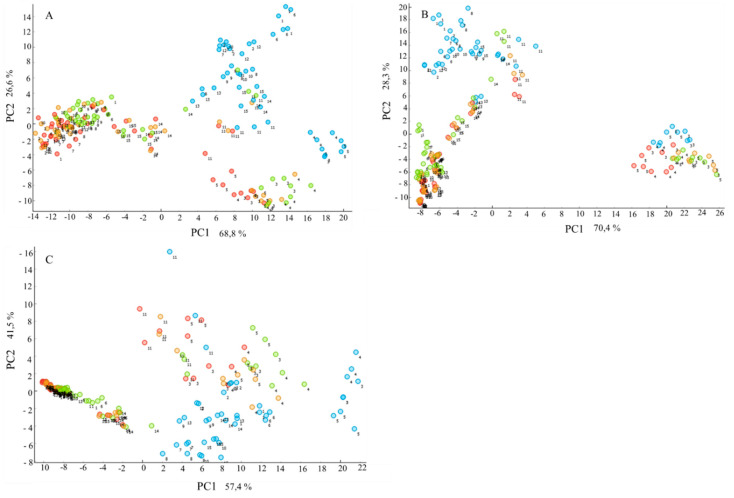
Principal component analysis (PCA) scatter plot of (**A**) lipid, (**B**) polysaccharide and (**C**) protein spectral regions of FTIR spectra of yeasts grown on media with C/N ratios: 10:1 (blue), 40:1 (green), 70:1 (orange) and 100:1 (red). Strains are labelled with numbers according to Table 1.

For identifying the biochemical profile of yeasts grown on different C/N ratios based on the obtained FTIR spectra, a principal component analysis (PCA) was applied. The PCA scatter plot shows that the C/N ratio was strongly affecting the lipid and protein profile in all yeasts, where the FTIR spectra of yeasts grown on the medium with a C/N ratio of 10:1 were grouped distinctly from the spectra of yeasts grown on other C/N ratios (Figure 5). This indicated that both total lipid content and fatty acid profile of accumulated TAGs in yeasts cells obtained from a C/N ratio of 10:1 medium was significantly different from yeasts grown on other ratios (Figure 5A,C). Further, some strain- and species-specific differences in lipid and protein profiles were observed from FTIR spectra. Thus, lipid and protein FTIR profiles for strains of genus *Metchnikowia* obtained from all types of media, and strains of species *S. salmonicolor* and *S. cererevisiae* grown on C/N ratios of 40:1, 70:1 and 100:1 were clearly different from the others (Figure 5A,C). These results correlate well with the obtained reference fatty acid profile data, where strains of *Metchnikowia*a and *S. salmonicolor* and *S. cerereviseae* differed from the others in the content of palmitic, palmitoleic, and polyunsaturated fatty acids (Figure 3). The polysaccharide profile was not affected by the C/N ratio in *Ascomycetes* yeasts, while significant differences were observed for *Basidiomycetes* yeasts (Figure 5B). The polysaccharide profile of strains from genus *Metchnikowia* and species *S. salmonicolor* differed significantly from other strains (Figure 5B).

### 3.5. Impact of Extracellular Osmolarity on Lipid and β-Glucans Coproduction

In this study, we used sodium chloride (NaCl) at six different concentrations (0.2; 0.5; 2; 5; 8 and 11%) to investigate the influence of different extracellular osmolarity levels on the production of β-glucans and coproduction of lipids and β-glucans. The three yeast strains *C. infirmominiatum* CCY 17-18-4, *P. rhodozyma* CCY 77-1-1 and *R. kratochvilovae* CCY 20-2-26, showing the highest β-glucans and a high lipid production in this study, were selected for the study. The *S. cerevisiae* CCY 19-6-4 strain was used as a control strain for β-glucan production. The growth media with C/N ratios of 40:1 and 70:1 supplemented by NaCl at all studied concentrations led to a decrease in the biomass yield for all yeast strains except for *S. cerevisiae* (Table 5, 0% NaCl). The biomass production for *S. cerevisiae* under low osmolarity conditions (0.2 and 0.5% of NaCl) in C/N ratio growth media with C/N ratios of 40:1, 70:1 and 100:1 was higher or very similar to the standard conditions (Table 5, 0% NaCl), while high osmolarity resulted in a significant decrease of biomass (Table 5). Low extracellular osmolarity (0.2% of NaCl) combined with a high C/N ratio resulted in an increase or no change in the biomass yield in comparison to the standard conditions (Table 5, 0% NaCl). The biomass yield was gradually decreasing with the increased amount of NaCl in the media. A high concentration of NaCl combined with a high C/N ratio resulted in the lowest biomass production which was possibly due to the elevated levels of osmolarity caused by both high NaCl and glucose content in the medium (Table 5). The strain *P. rhodozyma* CCY 77-1-1 showed the highest sensitivity to the applied osmotic stress, and its growth was highly inhibited in the presence of NaCl 2% and higher (Table 5).

The effect of extracellular osmolarity on the total glucan, β- and α-glucan content in yeast cells was strain-specific and differed depending on the level of osmolarity and the C/N ratio used (Table 6). Thus, low (0.2 and 0.5% of NaCl) and in some cases moderate (2 and 5%) levels of osmolarity combined with C/N ratios of 40:1, 70:1 and 100:1 led to an increase in the total glucan and β-glucan content in comparison to the reference conditions of 0% NaCl (Table 6). For example, the addition of 0.2% NaCl caused an increase in β-glucan production up to 32.15 ± 0.81 (*w*/*w*) in *C. infirmominiatum* CCY 17-18-4 (Table 6), that is about 21% more than in the standard conditions (Table 6, 0% NaCl) and higher than for the control strain *S.cerevisiae* CCY 21-4-102 (Table 6). In the case of strain *P. rhodozyma* CCY 77-1-1, β-glucan content was increased at all osmolarity levels (Table 6) when combined with C/N ratios of 40:1, 70:1 and 100:1, with the high yield obtained of 2.80 g/L of β-glucans at C/N 100:1 and 0.2% NaCl (*w*/*w*), while it showed an inhibiting effect on production of β-glucans in other carotenogenic yeast strains (Table 6). The β-glucan content was 1.98 g/L for *S. cerevisiae* CCY 21-4-102 at C/N 100:1 and 0.2% NaCl (*w*/*w*), which is higher than at standard conditions without salt.

High extracellular osmolarity (8 and 11% *w/v*) resulted in a decrease of lipid accumulation for all yeast strains in comparison to the standard growth conditions (Table 7, 0% NaCl), except for strain *S. cerevisiae* CCY 21-4-102, which did not show any significant changes in the lipid content when NaCl was added to the media (Table 7). Interestingly, yeast cells exposed to relatively moderate amounts of NaCl (2, 5% *w/v*) showed a slight increase in lipid accumulation for most of the studied yeast strains (Table 7).

## 4. Discussion

The simultaneous accumulation of multiple products is attractive for economically sustainable microbial biotechnology. Over the decades, the *Saccharomyces cerevisiae* is the only yeast strain used in industry for β-glucan production. Although the presence of β-1,3-glucan synthase has been identified in all fungal phyla (with the exception of *Microsporidia*), the carotenogenic *Basidiomycetes* yeast represents unexplored potential in biotechnological β-glucan production along with the coproduction of lipids [36,37].

In the present study, the screening of eleven carotenogenic *Basidiomycetes* yeasts for simultaneous β-glucan and lipid production and evaluating them as potential alternative sources of β-glucans was performed in a high-throughput set-up by utilizing high-throughput micro-cultivation in Duetz-MTPS [15,22,23]. Due to the fact that glucan production is usually performed in a low C/N ratio media [5], while lipid accumulation in yeasts is triggered by a high C/N ratio and depletion of a nitrogen source [38], media with both low and high C/N ratios were used for the screening. Further, since β-glucans are important structural components of the cell wall, applying osmotic stress could potentially have a significant effect on their production. In addition, it is well known that any variations in extracellular osmolarity influence the cell volume, and therefore, the concentration of intracellular macromolecules [39,40]. Thus, differences in osmolarity could potentially affect lipid accumulation in yeast cells. Recently, several studies reported the influence of extracellular osmolarity on β-glucans’ biosynthesis in *Saccharomyces cerevisiae* where a decrease in β-glucan content at higher osmolarity was detected [41,42]. Therefore, in our study we used different amounts of NaCl to investigate the effect of osmolarity on the coproduction of lipids and β-glucans in *Basidiomycetes* yeast. To the authors’ knowledge this is one of the first studies reporting evaluation of coproduction lipids and β-glucans in various carotenogenic yeasts.

As a cell wall polysaccharide component, β-glucan content can account for about 10% up to 30% of the DCW and the optimization of culture conditions to achieve high cell mass plays the crucial role in biotechnological β-glucan production [43]. Several researchers reported the screening of yeast strains, metabolic engineering, and optimization of different cultivation parameters: harvesting time, media composition, osmotic stress, for achieving high cell mass, and consequently high β-glucan yield [41,42,43,44,45,46,47,48,49,50,51]. In the literature it was reported quite different yields for β-glucan and biomass production. According to the authors’ knowledge, one of the highest reported β-glucan yields for small-scale screening of the *S. cerevisiae* strain was 2.076 g/L [42] which is higher than our results for *S. cerevisiae* CCY 21-4-102 (1.98 g/L of β-glucan, C/N 100, 0.2% NaCl *w*/*w*), while it is much lower when compared to the results for some *Basidiomycetous* yeast (*C. infirmominiatum* CCY 17-18-4, *R. kratochvilovae* CCY 20-2-26 and *P. rhodozyma* CCY 77-1-1 at high C/N ratio) reported in this study, where the β-glucan yield was in a range from 2.43 to 3.15 g/L. Some authors report β-glucan yield from 0.35 to 15% *w*/*w* for *S. cerevisiae* [52,53] and 25% *w*/*w* for *R. mucilaginosa* [5], that is lower compared to our results for *S. cerevisiae* CCY 20-4-102, and *C. infirmominiatum* CCY 17-18-4. Results on β-glucan yield reported by Valasquez et al. (2015) are higher than for *R. mucilaginosa* strains evaluated in our study which could indicate strain-specific β-glucan biosynthesis. Another alternative yeast producer of β-glucans, reported in the literature, is the yeast *Candida utilis* [54,55], which was able to produce 5.3 g/L of β-glucans when cultured on waste potato juice water supplemented with glycerol in a 5L bioreactor and 3.5 g/L when cultured in the shake flasks [55], that is comparable to our results for *C. infirmominiatum* CCY 17-18-4, which accumulated 3.15 g/L of β-glucans along with a high amount of lipids (38.21% DCW).

It has been previously shown that the C/N ratio is the key feature significantly affecting the accumulation of carbon-based intracellular metabolites (carbohydrates, lipids, pigments, etc.) [3,56,57,58]. Although there are many reports published on how C/N ratio influenced accumulation of lipids or carotenoids in *Basidiomycetes* yeast [3,7], the effect on β-glucan biosynthesis has not yet been investigated. In our study we observed that C/N ratio increased biomass production, with the highest yield observed for *C.infirmominiatum* CCY 17-18-4, which also showed the highest β-glucan yield at the C/N ratio 100:1 (3.15 g/L of β-glucan). Our study is reporting for the first time that *Cystofilobasidium* yeast can be considered as a potential alternative β-glucan source.

The decrease in β-glucan production for the *Cystofilobasidium* strains, as well as strains *R. toruloides* CCY 62-2-4, *R, kratochvilovae* CCY 20-2-26, and *P. rhodozyma* CCY 77-1-1 occurred under high C/N ratios and it can be explained by the increased accumulation of lipids which increase from 23 to 38% DCW. While under the condition of low C/N ratio, yeast proliferation is triggered and subsequently formation of the cell wall and carbon is transformed into protein and cell wall components, while lipogenesis is supressed. For some yeast strains (*R. mucilaginosa* CCY 19-4-6 and CCY 20-9-7, *S. pararoseus* CCY 19-9-6 and *S. salmonicolor* CCY 19-4-25), the high amount of glucose (100:1 C/N) led to a decrease in biomass production and, thus, the β-glucan yield. This could be due to the osmotic pressure stress under high glucose concentration that negatively influenced the growth of these strains. Therefore, C/N ratio should be considered as an important optimization parameter when developing coproduction of lipids and β-glucans.

In the last part of the study, eight different osmolarity conditions were applied, that resulted in a decrease of both biomass (12.35 ± 1.21 g/L) and lipid yield (35.49 ± 3.35% of DCW), while it was observed an increase of β-glucan yield, but only at low NaCl addition (22.65 ± 1.19% of DCW; 2.80 g/L). The biomass and β-glucans increased also at *S. cerevisiae* CCY 20-4-102 (6.32 ± 0.40 g/L of biomass, and 31.32 ± 0.93% *w*/*w* of β-glucans at 0.2% NaCl; C/N 100:1). On the other hand, NaCl content higher than 5% (*w*/*w*) negatively affected the accumulation of β-glucans. The obtained results are in accordance with those previously published by Varelas et al. (2017), where the 6% NaCl (*w*/*w*) resulted in a decrease of the β-glucans.

It is well-known that due to the high activity of AMP-dependent isocitrate dehydrogenase, some carotenogenic *Basidiomycetes* yeasts are able to produce lipids up to 70% lipids/dry weight [38,59,60] after optimized fed-batch fermentation under controlled conditions. In our screening the highest lipid content of 47.27 ± 2.36% (*w*/*w*) was observed for strain *C. macerans* CCY 10-1-2. Strains *C. informiniatum* CCY 17-18-4 and *R. kratochvilovae* CCY 20-2-26 also showed a high lipid content of 38.21 and 37.72% *w*/*w* along with the high β-glucan yield, which also was the highest among the studied *Basidiomyces* yeast. Thus, it could be concluded that these two strains have great potential for the future coproduction of lipids and β-glucan in a scaled-up strategy.

The highest amount of MUFA (49% from all fatty acids) was detected for the strain *S. metaroseus* CCY 19-6-20, which showed also one of the highest total lipid (45.13 ± 2.56% DCW) and biomass yields (15.11 ± 0.93 g/L) together with the β-glucan production up to 15% of DCW (2.27 g/L). Thus, *S. metaroseus* CCY 19-6-20 could be considered as a promising candidate for biofuel production, since MUFA is a desired oil component for biodiesel production providing low temperature fluidity and oxidative stability [61] with the concomitant β-glucan coproduction. Noncarotenogenic *Metschnikowia* phylum did not accumulate more than 10% of lipids, while it has been reported that it can produce up to 40% *w*/*w* [14], that could be due to the higher temperature and shorter cultivation time used in our study in comparison to the previously reported 15 °C and 14 days [14].

By achieving high biomass production, we can obtain a high β-glucan yield. As it was previously reported, the high cell density (biomass yield over 50 g/L) can be achieved with an optimized fed-batch strategy in fermenters with fully controlled conditions [43]. As for *Saccharomyces cerevisiae*, the highest biomass yield of 187.63 g/L was reported by Vu and Kim (2009), while the same biomass yield (185 g/L) was also obtained for carotenogenic yeast strain enriched with high lipid production (40% of DCW) reported by Pan et al. 1986. According to the literature, the biomass, lipid, and β-glucan yield can be optimized by the same strategy. Therefore, choosing *Basidiomycetes* yeast *C. infirmominiatum* CCY 17-18-4, *R. kratochvilovae* CCY 20-2-26, *P. rhodozyma* CCY 77-1-1, or *S. metaroseus* CCY 19-6-20 as promising candidates for the concomitant production of lipids and β-glucans, would achieve a sustainable coproduction process. Taking into consideration the increasing demand for β-glucan and lipids, carotenogenic *Basidiomycetes* yeast can be considered as an innovative source of these nutrients.

## 5. Conclusions

By applying a high-throughput screening approach, several carotenogenic *Basidiomycetes* yeast strains were identified as new sources of β-glucans, where the most promising results were obtained for strains *C. infirmominiatum* CCY 17-18-4, *R. kratochvilovae* CCY 20-2-26 and *P. rhodozyma* CCY 77-1-1. Further, several yeast strains showed high coproduction potential, for example strain *C. infirmominiatum* CCY 17-18-4 was able to accumulate 20.73% (*w*/*w*) of β-glucans and 38.21% (*w*/*w*) of lipids, the strain *R. kratochvilovae* CCY 20-2-26 was able to accumulate 38.21% (*w*/*w*) of lipids and 20.73% of β-glucans, accompanied by a high biomass yield (15.19 g/L). It was observed that C/N ratio and extracellular osmolarity could influence biomass yield and production of lipids and β-glucans. An increase in the C/N ratio led to an increase in biomass, lipid and β-glucan production for several yeast strains, while osmolarity had a negative effect on the biomass and lipid production but positively affected β-glucan production.

## Figures and Tables

**Table 1:** List of the yeast strains used in this study.

Number	Yeast Strain	Phylum	Strain Collection Number
1	*Cystofilobasidium infirmominiatum*	*Basidiomycetes*	CCY 17-18-4
2	*Cystofilobasidium macerans*	*Basidiomycetes*	CCY 10-1-2
3	*Metschnikowia pulcherrima*	*Ascomycetes*	CCY 29-2-149
4	*Metschnikowia pulcherrima*	*Ascomycetes*	CCY 29-2-147
5	*Metschnikowia pulcherrima*	*Ascomycetes*	CCY 29-2-129
6	*Phaffia rhodozyma*	*Basidiomycetes*	CCY 77-1-1
7	*Rhodotorula kratochvilovae*	*Basidiomycetes*	CCY 20-2-26
8	*Rhodotorula mucilaginosa*	*Basidiomycetes*	CCY 19-4-6
9	*Rhodotorula mucilaginosa*	*Basidiomycetes*	CCY 20-9-7
10	*Rhodotorula toruloides*	*Basidiomycetes*	CCY 62-2-4
11	*Saccharomyces cerevisiae*	*Ascomycetes*	CCY 21-4-102
12	*Sporidiobolus metaroseus*	*Basidiomycetes*	CCY 19-6-20
13	*Sporidiobolus pararoseus*	*Basidiomycetes*	CCY 19-9-6
14	*Sporidiobolus salmonicolor*	*Basidiomycetes*	CCY 19-6-4
15	*Sporidiobolus salmonicolor*	*Basidiomycetes*	CCY 19-4-25

**Table 2:** Biomass yield for yeast grown in the media with low and high C/N ratios.

		Biomass Yield (g/L)			
Strain Name	CCY	C/N 10:1	C/N 40:1	C/N 70:1	C/N 100:1
*Cystofilobasidium infirmominiatum*	17-18-4	3.80 ± 0.30	8.37 ± 0.31	13.06 ± 0.43	15.19 ± 0.91
*Cystofilobasidium macerans*	10-1-2	3.63 ± 0.24	8.76 ± 0.27	13.34 ± 0.06	15.33 ± 1.16
*Metschnikowia pulcherrima*	29-2-149	3.57 ± 0.05	5.42 ± 0.06	5.82 ± 0.18	6.78 ± 0.19
*Metschnikowia pulcherrima*	29-2-147	3.32 ± 0.06	4.99 ± 0.07	5.66 ± 0.19	5.73 ± 0.10
*Metschnikowia pulcherrima*	29-2-129	3.69 ± 0.24	5.49 ± 0.25	6.55 ± 0.34	6.73 ± 0.32
*Phaffia rhodozyma*	77-1-1	2.67 ± 0.26	6.78 ± 0.24	10.98 ± 0.50	13.09 ± 1.01
*Rhodotorula kratochvilovae*	20-2-26	4.15 ± 0.68	10.23 ± 0.17	12.05 ± 0.29	10.11 ± 1.37
*Rhodotorula mucilaginosa*	19-4-6	4.57 ± 0.29	10.36 ± 0.26	11.34 ± 0.23	10.84 ± 0.35
*Rhodotorula mucilaginosa*	20-9-7	4.30 ± 0.18	9.80 ± 0.18	9.97 ± 0.49	8.90 ± 1.52
*Rhodotorula toruloides*	62-2-4	3.65 ± 0.10	8.70 ± 0.20	11.80 ± 0.61	11.70 ± 0.80
*Saccharomyces cerevisiae*	21-4-102	3.59 ± 0.09	5.25 ± 0.36	5.32 ± 0.74	5.94 ± 0.56
*Sporidiobolus metaroseus*	19-6-20	3.67 ± 0.25	8.88 ± 0.05	13.42 ± 0.12	15.11 ± 0.93
*Sporidiobolus pararoseus*	19-9-6	3.83 ± 0.44	6.81 ± 1.30	10.88 ± 1.15	10.80 ± 1.88
*Sporidiobolus salmonicolor*	19-6-4	2.99 ± 0.15	6.30 ± 0.15	6.55 ± 0.31	8.24 ± 1.09
*Sporidiobolus salmonicolor*	19-4-25	3.49 ± 0.15	5.85 ± 0.27	5.88 ± 0.50	5.86 ± 0.36

**Table 3:** Total glucans, α- and β-glucan content (% of cell dry weight (CDW) in yeast grown in the media with low and high C/N ratios.

Strain		C/N 10:1	C/N 40:1	C/N 70:1	C/N 100:1
*C. infirmominiatum* CCY 17-18-4	Total glucans	26.04 ± 0.80	30.15 ± 3.21	24.78 ± 1.64	23.73 ± 2.25
	α-glucans	3.33 ± 0.39	4.81 ± 1.81	4.03 ± 0.94	3.01 ± 0.36
	β-glucans	22.72 ± 0.41	25.34 ± 3.79	20.75 ± 1.33	20.73 ± 1.39
*C. macerans* CCY 10-1-2	Total glucans	26.15 ± 1.38	23.61 ± 2.05	18.53 ± 2.08	16.32 ± 1.14
	α-glucans	2.68 ± 0.37	5.19 ± 3.30	5.08 ± 3.30	5.02 ± 3.25
	β-glucans	23.43 ± 1.60	18.42 ± 2.64	13.45 ± 4.42	11.31 ± 3.34
*M. pulcherrima* CCY 29-2-149	Total glucans	15.42 ± 0.90	18.26 ± 0.91	21.09 ± 0.94	21.34 ± 1.22
	α-glucans	0.29 ± 0.12	0.57 ± 0.20	0.64 ± 0.18	1.30 ± 0.31
	β-glucans	15.13 ± 1.02	17.69 ± 0.79	20.45 ± 0.86	20.04 ± 1.12
*M. pulcherrima* CCY 29-2-147	Total glucans	18.41 ± 1.55	19.84 ± 1.23	21.70 ± 1.56	21.73 ± 1.21
	α-glucans	0.35 ± 0.04	0.42 ± 0.04	0.77 ± 0.26	0.64 ± 0.11
	β-glucans	18.06 ± 1.54	19.42 ± 1.09	20.93 ± 1.80	21.09 ± 0.69
*M. pulcherrima* CCY 29-2-127	Total glucans	16.84 ± 1.08	21.13 ± 1.25	21.81 ± 1.56	23.25 ± 1.21
	α-glucans	0.47 ± 0.29	0.52 ± 0.19	1.28 ± 0.72	0.90 ± 0.59
	β-glucans	16.37 ± 1.12	20.61 ± 1.09	20.54 ± 1.10	22.35 ± 1.68
*P. rhodozyma* CCY 77-1-1	Total glucans	27.13 ± 0.88	24.93 ± 1.61	21.05 ± 1.74	20.35 ± 0.33
	α-glucans	2.61 ± 0.06	2.68 ± 0.75	2.20 ± 0.40	2.03 ± 0.28
	β-glucans	24.52 ± 0.85	22.28 ± 1.05	20.04 ± 0.18	18.55 ± 0.15
*R. kratochvilovae* CCY 20-2-26	Total glucans	23.18 ± 1.19	23.80 ± 1.12	21.84 ± 0.83	18.82 ± 0.29
	α-glucans	1.50 ± 0.34	1.04 ± 0.07	0.98 ± 0.11	1.23 ± 0.19
	β-glucans	21.68 ± 0.96	22.20 ± 1.04	21.43 ± 0.34	17.59 ± 0.55
*R. mucilaginosa* CCY 19-4-6	Total glucans	17.52 ± 0.64	14.60 ± 0.12	15.54 ± 1.04	15.99 ± 1.15
	α-glucans	0.87 ± 0.27	0.49 ± 0.09	0.66 ± 0.10	0.73 ± 0.04
	β-glucans	16.65 ± 0.67	14.11 ± 0.20	14.88 ± 1.08	15.26 ± 1.15
*R. mucilaginosa* CCY 20-9-7	Total glucans	18.18 ± 0.06	19.17 ± 0.67	19.91 ± 1.21	20.31 ± 0.95
	α-glucans	0.96 ± 0.32	0.98 ± 0.21	1.46 ± 0.72	1.18 ± 0.20
	β-glucans	17.22 ± 0.26	18.19 ± 0.65	18.45 ± 1.68	19.13 ± 1.13
*R. toruloides* CCY 62-2-4	Total glucans	19.26 ± 0.84	14.74 ± 0.25	11.75 ± 1.22	11.81 ± 0.58
	α-glucans	1.85 ± 0.30	1.97 ± 0.28	2.07 ± 0.42	1.78 ± 0.31
	β-glucans	17.41 ± 0.98	12.83 ± 0.37	9.67 ± 1.63	10.03 ± 0.72
*S.cerevisiae* CCY 21-4-102	Total glucans	20.54 ± 0.58	22.91 ± 2.03	26.21 ± 1.14	29.86 ± 3.11
	α-glucans	2.35 ± 0.38	2.35 ± 0.56	3.41 ± 0.80	2.90 ± 0.41
	β-glucans	18.19 ± 0.36	20.57 ± 1.54	22.80 ± 0.58	26.96 ± 2.90
*S. metaroseus* CCY 19-6-20	Total glucans	26.75 ± 2.59	22.77 ± 1.32	16.63 ± 1.00	17.68 ± 2.21
	α-glucans	2.60 ± 0.30	4.50 ± 0.36	2.62 ± 0.15	2.92 ± 0.23
	β-glucans	24.15 ± 2.89	18.27 ± 1.32	13.99 ± 1.04	14.76 ± 2.32
*S. pararoseus* CCY 19-9-6	Total glucans	14.30 ± 1.21	16.87 ± 1.91	15.58 ± 0.66	14.73 ± 0.79
	α-glucans	1.26 ± 0.45	2.41 ± 1.60	2.76 ± 1.88	3.51 ± 1.43
	β-glucans	13.04 ± 0.79	14.46 ± 3.13	12.81 ± 2.20	11.23 ± 1.72
*S. salmonicolor* CCY 19-6-4	Total glucans	12.90 ± 1.25	17.12 ± 0.92	17.96 ± 0.50	18.95 ± 2.00
	α-glucans	1.32 ± 0.28	2.28 ± 0.74	2.55 ± 0.57	2.43 ± 0.38
	β-glucans	11.58 ± 1.03	14.83 ± 1.32	15.41 ± 0.88	16.52 ± 2.28
*S. salmonicolor* CCY 19-4-25	Total glucans	15.04 ± 0.67	14.10 ± 0.31	14.95 ± 0.63	17.16 ± 1.73
	α-glucans	1.48 ± 0.14	2.35 ± 0.66	2.67 ± 0.76	2.84 ± 0.57
	β-glucans	13.56 ± 0.55	11.75 ± 0.59	12.28 ± 1.29	14.32 ± 2.30

**Table 4:** Peaks assignment for the FTIR spectra of yeast.

Peak №	Wavenumber	Peak Assignment	References
1	3010	= C-H stretching in lipids	[32]
2	2947	-C-H (CH_3_) stretching in lipids and hydrocarbons	[32]
3	2925	-C-H (CH_2_) stretching	[33]
4	2855	CH_2_/CH_3_ stretching in lipids and hydrocarbons	[32]
5	1745	C = O ester bond stretching in lipids, esters and polyesters	[33]
6	1680–1630	-C = O stretching, α-Helix amide I in proteins	[34]
7	1530–1560	N-H bending and C-N stretching, amide II in proteins	[34]
8	1465	CH_2_/CH_3_ stretching in lipids	[32]
9	1377	-C-H (CH_3_) bending (sym) in lipids	[35]
10	1265	–P = O stretching of phosphodiesters	[35]
11	1200−1100	C-O-C/C-O stretching in polysaccharides	[35]

**Table 5:** The effect of different osmolarity levels on biomass yield in strains *C. infirmominiatum* CCY 17-18-4 (1), *P. rhodozyma* CCY 77-1-1 (6), *R. kratochvilivae* CCY 20-2-26 (7), *S. cerevisiae* CCY 21-4-102 (11).

Strain	C/N	0% NaCl	0.2% NaCl	0.5% NaCl	2% NaCl	5% NaCl	8% NaCl	11% NaCl
1	40	8.37 ± 0.31	7.44 ± 0.04	7.38 ± 0.19	7.08 ± 0.11	6.38 ± 0.07	5.93 ± 0.07	4.81 ± 0.61
	70	13.06 ± 0.43	11.40 ± 0.10	11.47 ± 0.35	10.77 ± 0.07	10.28 ± 0.07	9.13 ± 0.49	4.54 ± 0.84
	100	15.19 ± 0.91	15.25 ± 0.39	14.88 ± 0.11	11.40 ± 2.84	11.33 ± 0.05	8.90 ± 0.59	4.73 ± 0.24
6	40	6.78 ± 0.24	5.62 ± 0.41	5.56 ± 0.08	2.24 ± 0.16	1.05 ± 0.14	0.70 ± 0.16	0.68 ± 0.07
	70	10.98 ± 0.50	8.86 ± 0.61	8.69 ± 0.27	2.59 ± 0.21	1.06 ± 0.09	0.87 ± 0.16	0.64 ± 0.10
	100	13.09 ± 1.01	12.35 ± 1.21	11.11 ± 1.48	2.73 ± 0.19	1.00 ± 0.03	0.78 ± 0.23	0.66 ± 0.13
7	40	10.23 ± 0.17	9.04 ± 0.31	8.57 ± 0.30	8.44 ± 0.07	7.52 ± 0.10	5.71 ± 0.25	3.71 ± 0.09
	70	12.05 ± 0.29	11.76 ± 0.81	11.26 ± 0.73	10.70 ± 0.04	8.47 ± 0.08	5.80 ± 0.46	3.90 ± 0.39
	100	10.11 ± 1.37	11.52 ± 0.74	10.83 ± 0.75	10.48 ± 0.04	8.24 ± 0.06	5.69 ± 0.42	3.69 ± 0.47
11	40	5.25 ± 0.36	5.45 ± 0.11	5.26 ± 0.20	5.03 ± 0.07	3.25 ± 0.04	2.71 ± 0.16	1.78 ± 0.13
	70	5.32 ± 0.74	5.94 ± 0.14	5.70 ± 0.31	4.97 ± 0.06	3.82 ± 0.05	3.03 ± 0.29	1.69 ± 0.04
	100	5.94 ± 0.56	6.32 ± 0.40	5.71 ± 0.47	4.95 ± 0.09	4.00 ± 0.03	3.04 ± 0.28	1.57 ± 0.06

**Table 6:** The effect of different osmolarity levels on glucan production in strains *C. infirmominiatum* CCY 17-18-4 (1), *P. rhodozyma* CCY 77-1-1 (6), *R. kratochvilivae* CCY 20-2-26 (7), *S. cerevisiae* CCY 21-4-102 (11).

Strain	C/N	% *w*/*w*	0% NaCl	0.2% NaCl	0.5% NaCl	2% NaCl	5% NaCl	8% NaCl	11% NaCl
1	40	Total glucan	30.15 ± 3.21	35.65 ± 0.86	34.10 ± 1.91	32.87 ± 1.75	22.71 ± 1.67	21.65 ± 0.97	20.80 ± 1.56
		Alpha-glucan	4.81 ± 1.81	3.50 ± 0.40	3.55 ± 0.39	3.07 ± 0.32	2.96 ± 0.22	3.03 ± 0.48	2.63 ± 0.43
		Beta-glucan	25.34 ± 3.79	32.15 ± 0.81	30.55 ± 1.84	29.80 ± 1.43	19.75 ± 1.87	18.62 ± 0.67	18.17 ± 1.55
	70	Total glucan	24.78 ± 1.64	27.15 ± 1.26	25.42 ± 0.38	21.11 ± 0.44	23.33 ± 1.07	19.72 ± 0.56	21.07 ± 0.96
		Alpha-glucan	4.03 ± 0.94	3.16 ± 0.21	3.11 ± 0.30	2.79 ± 0.10	3.87 ± 1.16	2.70 ± 0.12	2.79 ± 0.77
		Beta-glucan	20.75 ± 1.33	23.99 ± 1.06	22.32 ± 0.63	18.32 ± 0.52	19.46 ± 1.54	17.03 ± 0.66	18.28 ± 1.23
	100	Total glucan	23.73 ± 2.25	21.83 ± 0.27	21.64 ± 1.47	23.16 ± 0.45	19.95 ± 1.58	17.96 ± 0.82	21.52 ± 2.16
		Alpha-glucan	3.01 ± 0.36	3.04 ± 0.29	3.41 ± 0.36	4.42 ± 1.29	3.04 ± 0.18	3.20 ± 0.03	3.11 ± 0.77
		Beta-glucan	20.73 ± 1.39	18.78 ± 0.49	18.24 ± 1.11	18.73 ± 1.71	16.91 ± 1.40	14.76 ± 0.82	18.40 ± 2.02
6	40	Total glucan	24.93 ± 1.61	31.69 ± 1.97	32.51 ± 0.51	24.47 ± 1.76	27.75 ± 0.99	29.05 ± 0.46	26.25 ± 0.87
		Alpha-glucan	2.68 ± 0.75	4.37 ± 2.58	3.25 ± 0.52	3.44 ± 0.17	3.05 ± 0.45	3.25 ± 0.98	2.72 ± 0.56
		Beta-glucan	22.28 ± 1.05	27.32 ± 4.55	29.26 ± 1.02	21.03 ± 1.93	24.70 ± 0.60	25.80 ± 1.26	23.53 ± 1.44
	70	Total glucan	21.05 ± 1.74	24.27 ± 2.24	24.30 ± 0.97	26.60 ± 2.10	27.18 ± 0.81	25.65 ± 0.74	24.27 ± 1.37
		Alpha-glucan	2.20 ± 0.40	2.42 ± 0.35	2.71 ± 0.32	3.05 ± 0.24	2.69 ± 0.30	3.24 ± 0.66	2.46 ± 0.89
		Beta-glucan	20.04 ± 0.18	21.85 ± 2.07	21.59 ± 0.65	23.55 ± 2.31	24.49 ± 0.96	22.41 ± 0.20	21.81 ± 0.69
	100	Total glucan	20.35 ± 0.33	24.97 ± 1.16	27.56 ± 1.29	24.29 ± 1.21	28.11 ± 0.87	26.74 ± 0.83	26.02 ± 1.06
		Alpha-glucan	2.03 ± 0.28	2.32 ± 0.35	2.73 ± 0.57	3.09 ± 0.13	2.58 ± 0.50	2.87 ± 0.36	2.81 ± 0.88
		Beta-glucan	18.55 ± 0.15	22.65 ± 1.19	24.83 ± 1.86	21.20 ± 1.20	25.53 ± 1.28	23.87 ± 0.46	23.21 ± 1.17
7	40	Total glucan	23.80 ± 1.12	24.13 ± 2.17	23.53 ± 2.53	17.08 ± 1.09	13.29 ± 0.96	14.67 ± 0.67	12.23 ± 1.90
		Alpha-glucan	1.04 ± 0.07	1.25 ± 0.28	1.19 ± 0.11	1.13 ± 0.26	0.91 ± 0.14	1.19 ± 0.14	0.84 ± 0.23
		Beta-glucan	22.20 ± 1.04	22.88 ± 2.37	22.34 ± 2.53	15.95 ± 0.85	12.38 ± 1.04	13.47 ± 0.61	11.39 ± 1.67
	70	Total glucan	21.84 ± 0.83	18.97 ± 1.45	20.32 ± 0.10	16.41 ± 1.00	11.81 ± 1.09	14.48 ± 1.85	14.84 ± 2.38
		Alpha-glucan	0.98 ± 0.11	1.12 ± 0.33	0.83 ± 0.38	0.93 ± 0.31	0.68 ± 0.13	0.90 ± 0.24	1.14 ± 0.19
		Beta-glucan	21.43 ± 0.34	17.84 ± 1.20	19.49 ± 0.44	15.48 ± 1.30	11.14 ± 1.05	13.58 ± 1.62	13.69 ± 2.20
	100	Total glucan	18.82 ± 0.29	20.36 ± 0.46	18.18 ± 1.00	13.03 ± 0.53	12.08 ± 0.67	15.68 ± 0.29	9.42 ± 0.38
		Alpha-glucan	1.23 ± 0.19	1.20 ± 0.24	1.07 ± 0.24	0.68 ± 0.11	0.59 ± 0.07	1.05 ± 0.31	0.81 ± 0.20
		Beta-glucan	17.59 ± 0.55	19.16 ± 0.64	17.11 ± 0.78	12.35 ± 0.63	11.49 ± 0.63	14.63 ± 0.31	8.62 ± 0.34
11	40	Total glucan	22.91 ± 2.03	30.40 ± 0.72	29.87 ± 1.69	23.95 ± 2.51	23.76 ± 2.93	19.24 ± 1.12	16.31 ± 1.29
		Alpha-glucan	2.35 ± 0.56	1.49 ± 0.18	1.31 ± 0.35	2.05 ± 0.32	1.21 ± 0.17	0.67 ± 0.21	0.35 ± 0.13
		Beta-glucan	20.57 ± 1.54	28.91 ± 0.62	28.56 ± 1.37	21.90 ± 2.81	22.55 ± 2.77	18.57 ± 0.96	15.93 ± 1.45
	70	Total glucan	26.21 ± 1.14	29.16 ± 1.40	27.29 ± 0.36	31.08 ± 2.42	25.83 ± 2.12	18.07 ± 0.45	16.94 ± 2.13
		Alpha-glucan	3.41 ± 0.80	1.69 ± 0.39	1.54 ± 0.42	2.19 ± 0.32	1.50 ± 0.10	0.52 ± 0.20	0.58 ± 0.16
		Beta-glucan	22.80 ± 0.58	27.47 ± 1.03	25.75 ± 0.57	28.89 ± 2.40	24.33 ± 2.15	17.56 ± 0.58	16.36 ± 2.24
	100	Total glucan	29.86 ± 3.11	33.38 ± 0.93	31.55 ± 2.43	30.06 ± 2.11	24.32 ± 0.92	21.67 ± 0.29	15.59 ± 2.03
		Alpha-glucan	2.90 ± 0.41	2.06 ± 0.19	1.13 ± 0.47	1.75 ± 0.31	1.03 ± 0.09	1.05 ± 0.32	0.50 ± 0.14
		Beta-glucan	26.96 ± 2.90	31.32 ± 0.93	30.42 ± 2.02	28.31 ± 2.27	23.29 ± 0.84	20.62 ± 0.10	15.10 ± 1.92

**Table 7:** The effect of different osmolarity levels on the lipid production (% of DCW) in strains *C. infirmominiatum* CCY 17-18-4 (1), *P. rhodozyma* CCY 77-1-1 (6), *R. kratochvilivae* CCY 20-2-26 (7), *S. cerevisiae* CCY 21-4-102 (11).

Strain	C/N	0% NaCl	0.2% NaCl	0.5% NaCl	2% NaCl	5% NaCl	8% NaCl	11% NaCl
1	40	23.28 ± 1.82	19.66 ± 1.58	18.64 ± 2.13	24.39 ± 0.67	29.07 ± 1.85	26.94 ± 1.28	19.50 ± 1.12
	70	33.65 ± 2.12	25.58 ± 1.80	25.57 ± 2.15	30.88 ± 0.88	32.97 ± 0.94	36.27 ± 3.05	24.87 ± 0.98
	100	38.21 ± 3.25	39.85 ± 2.62	36.06 ± 4.36	35.22 ± 4.14	35.26 ± 2.12	31.31 ± 1.74	25.25 ± 2.24
6	40	24.94 ± 1.20	22.08 ± 1.08	21.65 ± 17	27.95 ± 2.10	31.40 ± 1.69	16.57 ± 0.65	20.40 ± 0.60
	70	36.82 ± 0.58	31.84 ± 0.31	35.13 ± 0.68	29.55 ± 1.85	36.64 ± 3.93	22.48 ± 4.36	19.38 ± 2.14
	100	38.00 ± 0.68	35.49 ± 3.35	31.78 ± 5.69	30.04 ± 0.43	36.32 ± 2.14	20.16 ± 1.22	25.35 ± 1.97
7	40	32.85 ± 0.94	26.13 ± 3.85	26.62 ± 0.97	29.62 ± 0.99	31.33 ± 0.54	29.67 ± 1.11	18.91 ± 1.47
	70	37.86 ± 2.56	27.66 ± 1.09	32.28 ± 3.04	35.04 ± 2.56	34.18 ± 2.29	31.40 ± 3.72	25.23 ± 2.20
	100	36.52 ± 0.66	31.82 ± 2.83	36.79 ± 4.17	31.35 ± 1.98	34.96 ± 2.23	30.09 ± 1.60	21.59 ± 1.49
11	40	7.34 ± 0.18	6.62 ± 0.90	8.39 ± 0.71	8.87 ± 1.28	7.03 ± 0.55	7.99 ± 0.41	9.14 ± 0.76
	70	8.54 ± 0.29	10.24 ± 0.21	10.48 ± 0.65	9.12 ± 0.67	10.99 ± 0.36	7.83 ± 0.88	12.09 ± 1.37
	100	9.20 ± 0.66	5.68 ± 0.19	8.67 ± 2.49	9.40 ± 0.87	11.73 ± 0.94	10.65 ± 0.45	13.14 ± 1.65
